# Phenolic compound abundance in Pak choi leaves is controlled by salinity and dependent on pH of the leaf apoplast

**DOI:** 10.1002/pei3.10039

**Published:** 2021-02-04

**Authors:** Philipp Meyer, Nadja Förster, Susanne Huyskens‐Keil, Christian Ulrichs, Christoph‐Martin Geilfus

**Affiliations:** ^1^ Faculty of Life Sciences Division of Controlled Environment Horticulture Humboldt‐Universität zu Berlin Berlin Germany; ^2^ Faculty of Life Sciences Division Urban Plant Ecophysiology Humboldt‐Universität zu Berlin Berlin Germany

**Keywords:** apoplastic pH, chloride, flavonoids, Pak choi, phenolic acids, salt stress

## Abstract

Onset of salinity induces the pH of the leaf apoplast of Pak choi transiently to increase over a period of 2 to 3 hr. This pH event causes protein abundances in leaves to increase. Among them are enzymes that are key for the phenylpropanoid pathway. To answer the questions whether this short‐term salt stress also influences contents of the underlying phenylpropanoids and for clarifying as to whether the apoplastic pH transient plays a role for such a putative effect, Pak choi plants were treated with 37.5 mM CaCl_2_ against a non‐stressed control. A third experimental group, where the leaf apoplast of plants treated with 37.5 mM CaCl_2_, was clamped in the acidic range by means of infiltration of 5 mM citric acid/sodium citrate (pH 3.6), enabled validation of pH‐dependent effects. Microscopy‐based live cell imaging was used to quantify leaf apoplastic pH in planta. Phenolics were quantified shortly after the formation of the leaf apoplastic pH transient by means of HPLC‐DAD‐ESI‐MS. Results showed that different phenolic compounds were modulated at 150 and 200 min after the onset of chloride salinity. A pH‐independent reduction in phenolic acid abundance as well as an accumulation of phenolic acid:malate conjugates was quantified after 200 min of salt stress. However, at 150 min after the onset of salt stress, flavonoids were significantly reduced by salinity in a pH‐dependent manner. These results provided a strong indication that the pH of the apoplast is a relevant component for the short‐term metabolic response to chloride salinity.

## Graphical abstract

We show that the transient alkalinization is a remote effect of chloride stress at the roots that, in turn, modulates abundances of phenols in leaves. For the first time, a function downstream of the transient pH increase was identified.

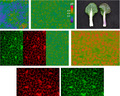

**Abbreviations**
ABA: abscisic acid
CAD: cinnamyl alcohol dehydrogenase
CAAD: caffeic acid derivative
CMA: caffeoyl malate
COA: coumarylquinic acid
CQA: caffeoylquinic acid
DW: dry weigth
FGL: feruloyl glucoside
FL: flavonoids
FMA: feruloyl malate
HFM: hydroxyferuloyl malate
IRG: Isorhamnetin‐3‐O‐glucoside
KCDG: Kaempferol‐3‐O‐caffeoyl diglucoside‐7‐O‐glucoside
KHDG: Kaempferol‐3‐O‐hydroxyferuloyl diglucoside‐7‐O‐glucoside
PA: phenolic acids
PAL: phenylalanine ammonia lyase
PAmC: phenolic acid:malate conjugates
pH_apo_
: pH of the apoplast
PPFD: photosynthetic photon flux density
*SEM*
: standard error of mean
SMA: Sinapoyl malate

## 1 INTRODUCTION

Salt stress is a common abiotic stress that is recognized for its multifaceted negative impact on plant growth. With more than 800 million hectares of salt‐affected land worldwide (Munns & Tester, 2008) and in respect of the ever growing demand for food and fodder crops, plant scientist are encouraged to develop a set of means to warrant food security.

Salinity affects plants in two ways. First, it reduces the osmotic potential of the soil solution, decreasing water and nutrient uptake by plants. This osmotic stress component acts fast (hours to days) and reduces the rates by which shoots, leaves and to a lesser degree roots expand (Bartels & Sunkar, 2005; Feng et al., 2018). Adaptions either comprises increased net rate of osmolyte deposition (e.g. hexoses, K^+^) or reduced cell expansion (Sharp et al., 1990). In other words, the plant struggles to take up water and strives to lower the cellular osmotic potential in order to facilitate water uptake (Zörb et al., 2019). In the longer term (days to month), excessive amounts of salt ions accumulate in the tissue. This second so‐called ion toxic phase is characterized by a dysfunctional photosynthetic machinery (Bose et al., 2017) that gives rise to the formation and accumulation of reactive oxygen species. These radicals damage plasma membranes, proteins or DNA by oxidation (Mittler, 2017). Excessive concentration of the salt ions in the cytosol is also suggested to inhibit activities of enzymes (Flowers et al., 2010). As reviewed by Munns and Tester (2008), ion‐specific salt‐tolerance is often acquired by at least one of the two strategies: (i) The exclusion of ions by roots, which avoids accumulation of ions in the shoot and (ii) the ability of leaf tissue to tolerate high ion concentrations by ion sequestration into the vacuole.

Overall, much is known about these mid to long‐term stress responses, that is, about the osmotic and the ionic phase, whereas information about the fast response that ensue minutes to hours after the onset of salt stress is scanty. There are a few studies on fast salinity‐induced changes of gene expression and proteome patterns, suggesting for rice that this early phase is indeed important for adaptation (time frame: 15 min to 6 hr; (Kawasaki et al., 2001; Roshandel & Flowers, 2009; Zhang et al., 2009)). Likewise, it was reported that changes of the gene and miRNA (Hernandez et al., 2020; Popova et al., 2008; Ueda et al., 2004) expression profile, as well as of the metabolome (Kim et al., 2007; Wang et al., 2009) coincide with the earliest phase of salt stress.

Another short‐term effect comprises changes in the pH of the leaf apoplast. Recently, we reported about changes of the apoplastic pH (pH_apo_) in leaves of *Vicia faba* that were formed at 20 min after plants were challenged by salinity at the root level. These pH changes were induced by the chloride‐component of NaCl salinity being able to modulate the (i) distribution (Geilfus et al., 2015) and (ii) gene expression (Geilfus et al., 2020) of the phytohormone abscisic acid (ABA), causing stomata to close. This transient alkalinization of the apoplast also induced changes of the leaf proteome that were detectable after 2 to 3 hr (Geilfus et al., 2017). Among the salt‐responsive proteins were enzymes of the branched phenylpropanoid pathway, namely phenylalanine ammonia lyases (PALs) and the cinnamyl alcohol dehydrogenase (CAD). Both are pivotal for the synthesis of polyphenols (e.g. flavonoids) (Winkel‐Shirley, 2002) and monolignols (Halpin et al., 1994) and are likely to be key enzymes for stress adaptations. It is not known whether these compounds are affected quantitatively during the onset of chloride salinity in a pH_apo_‐dependent mode.

This study was conducted in order to investigate whether a short‐term chloride salinity treatment modulates abundances of compounds that are synthesized by the phenylpropanoid pathway. Second, it aimed at clarifying the role of the leaf pH_apo_ for such putative responses. We studied this response in Pak choi [*Brassica campestris* L. ssp. chinensis (L.) Makino] because it is an important leafy vegetable for human nutrition that is rich in phenylpropanoids (Harbaum et al., 2007). By means of HPLC‐analyses of secondary metabolites, together with ratiometric real‐time *in‐planta* measurements of pH in intact plants, the present study demonstrates that salinity reduced flavonoid contents in a pH_apo_‐dependent manner, whereas changes of phenolic acids were found to be pH_apo_‐independent.

## 2 MATERIALS AND METHODS

### 2.1 Plant cultivation and experimental design

Pak choi [*Brassica campestris* L. ssp. chinensis (L.) Makino] was grown hydroponically in a Vötsch climate chamber (350 μmol/s m^−2^ PPFD; 14/10 hr day/night; 20/15°C; 60/50% humidity). Seeds were first soaked in CaSO_4_ solution (1 mM) for 2 days to facilitate germination. During germination, seeds were placed into quartz sand moistened with CaSO_4_. After 10 days of germination, seedlings were transferred to plastic pots containing nutrient solution. The nutrient solution had the following composition: 0.1 mM KH_2_PO_4_, 1.0 mM K_2_SO_4_, 0.2 mM KCl, 2.0 mM Ca(NO_3_)_2_, 0.5 mM MgSO_4_, 60 μM Fe‐EDTA, 10 μM H_3_BO_4_, 2.0 μM MnSO_4_, 0.5 μM ZnSO_4_, 0.2 μM CuSO_4_, 0.05 μM (NH_4_)_6_Mo_7_O_24_. The solution was changed every 2.5 days to avoid nutrient depletion. After 21 days, plants were divided into three experimental groups. Plants from the first group (*n* = 5) were stressed via addition of 37.5 mM CaCl_2_ to the roots. Plants from the second group (*n* = 5) were also stressed via addition of 37.5 mM CaCl_2_ to the roots. However, before the stress treatment was initiated, the leaf pH_apo_ of the fourth oldest leaf was clamped in the acid range via infiltration of 5 mM citric acid/sodium citrate (pH 3.6). Plants from the third group (*n* = 5) represent the non‐stressed controls (neither CaCl_2_ to the roots nor buffer infiltration). The fourth oldest leaf of plants of group 1 and 3 was infiltrated with water as a control to check the effects of the infiltration procedure. At 150 and 200 min after the addition of 37.5 mM CaCl_2_ to the roots, leaves were harvested, immediately shock frosted and freeze‐dried to determine the contents of flavonoids and phenolic acids. The leaf apoplastic pH was measured continuously every 10 min over the entire 200 min of the experiment using live cell imaging. Leaf chloride content was measured every 50 min. While pH_apo_ measurement was conducted non‐invasively via optical methods, measurements of leaf chloride content and metabolites were performed destructively, requiring a separate batch of plants for each time point. In the present work, all analyses were performed on the fourth oldest leaf (*n* = 5 biological replicates).

### 2.2 Inverse microscopy imaging

The leaf pH_apo_ was quantified *in planta* via apoplastic H^+^‐live‐imaging using a microscopy‐based ratiometric approach. In brief, the dextranated (10 kDa) pH‐sensitive dye Oregon Green 488 (Invitorgen GmbH, Darmstadt, Germany; dissolved in deionized water) was used as an apoplastic pH‐sensor. For this, it was infiltrated through the open stomata into the apoplast as described by Geilfus and Mühling (2011). Dye signals were detected with a fluorescence microscope (DMI6000B; Leica Microsystems, Wetzlar, Germany) using a dry objective (HCX PL FLUOTAR L, Leica Microsystems, Wetzlar, Germany) and a DFC‐camera. Dye was exposed for 25 mS at the excitation wavelengths of 440/20 (pH‐insensitive) and 495/10 nm (pH‐sensitive). Light emission at both channels was collect at 535/25 nm. As a measure of pH, the fluorescence ratio F_495_/F_440_ was calculated. Ratio values were converted into pH values via an in vivo calibration (Geilfus et al., 2014).

### 2.3 Analysis of leaf Cl^−^ contents

Leaf Cl^−^ contents were quantified in 15 mg dried leaf samples. For extraction, milled leaf samples were boiled for 5 min in 1.6 ml deionized water. After being cooled down on ice, the samples were centrifuged. The supernatant was collected and proteins were removed by chloroform precipitation. Hydrophobic compounds were removed by a passage through a C18‐E column (Strata, Phenomenex, Torrance, CA, USA). Purified samples were ready for the measurement of chloride concentration using ion chromatography (ICS 5000,Thermo‐Dionex, Sunnyvale, CA, USA) as described elsewhere (Geilfus et al., 2017).

### 2.4 Determination of flavonoids and phenolic acids

To analyze the profile of selected phenolic acids and flavonoids in the Pak choi leaves, 20 mg lyophilized powder was used for the extraction. Based on a method described by Förster et al. (2015), the leaf powder was extracted in 300 µl of 70% methanol (pH 4, acetic acid) for 15 min in ice water using sonification (Bandelin Sonorex). The pellet was re‐extracted twice with 300 µl of the extraction solvent for 10 min. After each extraction step, the samples were centrifuged for 5 min at 16,000 g (Thermo Scientific, Heraeus Megafuge X1R Centrifuge) at 4°C and the supernatants were combined. Supernatants were concentrated (vacuum concentrator, Thermo Scientific Savant SPD111V Concentrator, vacuum pump: Vacuubrand PC 3001 series, CVC3000) to near dryness, dissolved in 50% methanol, and filled up to 1 ml. The samples were shortly vortexed and centrifuged for 10 min at 16,000 g, filtered (Costar^®^ SpinX tubes), and transferred to HPLC vials. Phenolic acids and flavonoids in extracts were qualitatively and quantitatively analyzed by HPLC (Ultimate 3,000, Thermo Scientific). A volume of 10 µl extract was injected and separated using a 150 x 2.1 mm C16 column (AcclaimPA, 3 μm, Thermo Scientific). Two solvents were used for analysis: solvent A: H_2_O (0.5% formic acid), B: 40% acetonitrile. For separation, the following gradient program was used: 0–1 min: 0.5% B, 1–10 min: 0.5%–40% B, 10–12 min: 40% B, 12–18 min: 40%–80% B, 18–20 min: 80% B, 20–24 min: 80%–100% B, 24–30 min: 100% B, 30–34 min: 100%–0.5% B, and 34–39 min 0.5% B at a flow rate of 0.4 ml/min. The oven temperature was 35°C. Detection was carried out at 290 nm on a photodiode array detector. Phenolic acids and flavonoids were quantified against the internal standard 4‐methoxycinnamic acid (1 mM, Sigma Aldrich). Commercially available standards of single compounds were used as reference (chlorogenic acid, p‐coumaryl acid, trans‐ferulic acid, caffeic acid, caffeoyl malate, kaempferol 3‐glucoside, and quercetin 7‐O‐glucoside). Relative response factors were used to correct for absorbance difference. If a standard was not available, response factors of a compound with a similar chemical structure was used. Qualitative identification of compounds was carried out by retention time, specific UV‐spectra, and mass spectrometry ([M‐H]^−^), HPLC‐DAD‐ESI‐MS^3^).

### 2.5 Data evaluation

Statistical analyses were conducted in R (R Core Team, 2014) and figures were produced using the package ggplot2 (Wickham, 2009). Data are presented as mean ± standard error of mean (*SEM*). For each secondary plant metabolite and sampling time point the effect of treatment was determined by a one‐way ANOVA (α = 0.05). P‐values were adjusted according to the Bonferroni correction method for multiple comparisons. When effects were significant, Tukey's HSD test was performed to discriminate between treatment groups.

## 3 RESULTS

### 3.1 Ratiometric in planta monitoring of leaf apoplastic pH

**FIGURE 1:**
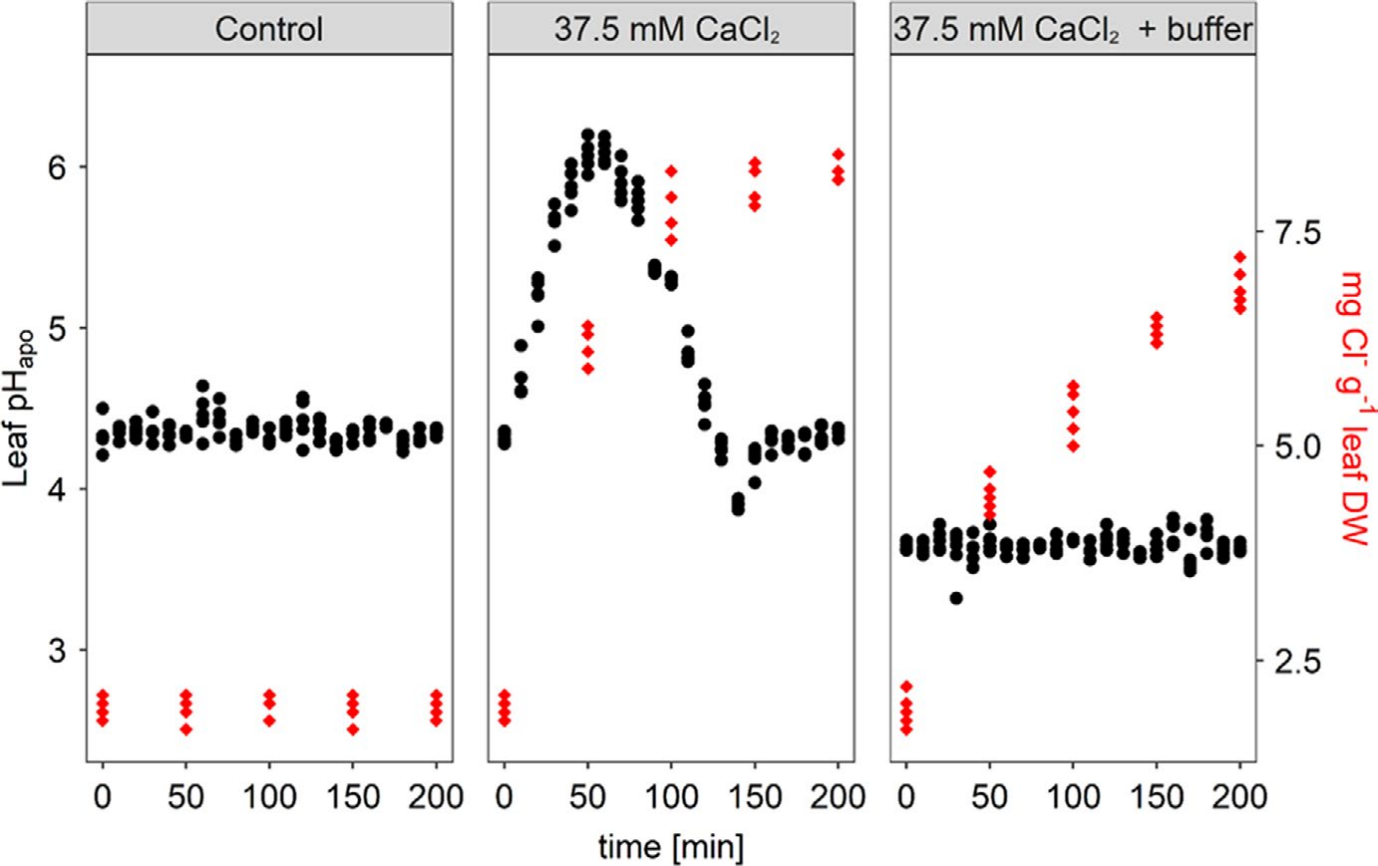
Treating Pak choi roots with 37.5 mM CaCl_2_ increased leaf chloride content (red diamonds) and modulated leaf apoplastic pH (black circles). Plants were either grown under non‐stressed conditions (Control) or under salt stress by addition of CaCl_2_ to the roots (37.5 mM CaCl_2_). An additional set of plants (37.5 mM CaCl_2_ + buffer) was introduced to a CaCl_2_–treatment in combination with infiltration of a pH buffer into the leaf apoplast (5 mM citric acid/sodium citrate at pH 3.6). Representative kinetics of five equivalent recordings of plants gained from independent experiments (*n* = 5 biological replicates)

The ratiometric pH quantification revealed a transient alkalinization of the leaf apoplast when intact Pak choi plants were treated with 37.5 mM CaCl_2_ at the root level. Starting from a pH_apo_ of 4.3, a rise occurred for 60 min, resulting in a maximum pH of 6.1. After 10 min of stagnation, the pH steadily decreased to 3.9 and ultimately stabilized at the initial pH of 4.3 (Figure 1). This transient alkalinization could be inhibited by the infiltration of a pH‐buffer (5 mM citric acid/sodium citrate) into the leaf apoplast prior to exposing roots to 37.5 mM CaCl_2_. However, this pH‐buffering lowered the pH_apo_ to 3.6. The pH_apo_ in the non‐stressed control groups was stable at 4.4 over the entire experiment (Figure 1).

### 3.2 Leaf chloride concentration

Under control conditions, Pak choi leaves contained 2 mg Cl^−^ g^−1^ dry weight (DW) (Figure 1; red kinetics). The addition of 37.5 mM CaCl_2_ to the roots induced a fast accumulation of Cl^−^. Already after 50 min, measurements revealed a significant increase to 6.2 mg Cl^−^ g^−1^ leaf DW, reaching a content of 8.3 mg Cl^−^ g^−1^ leaf DW after 200 min. The pH‐buffering of the leaf apoplast provoked a less steep increase in Cl^−^ compared to the non‐pH_apo_‐buffered experimental group that was stressed with CaCl_2_ , resulting in lower content of 6.9 mg Cl^−^ g^−1^ leaf DW after 200 min.

### 3.3 Quantification of flavonoid and phenolic acid derivatives

**FIGURE 2:**
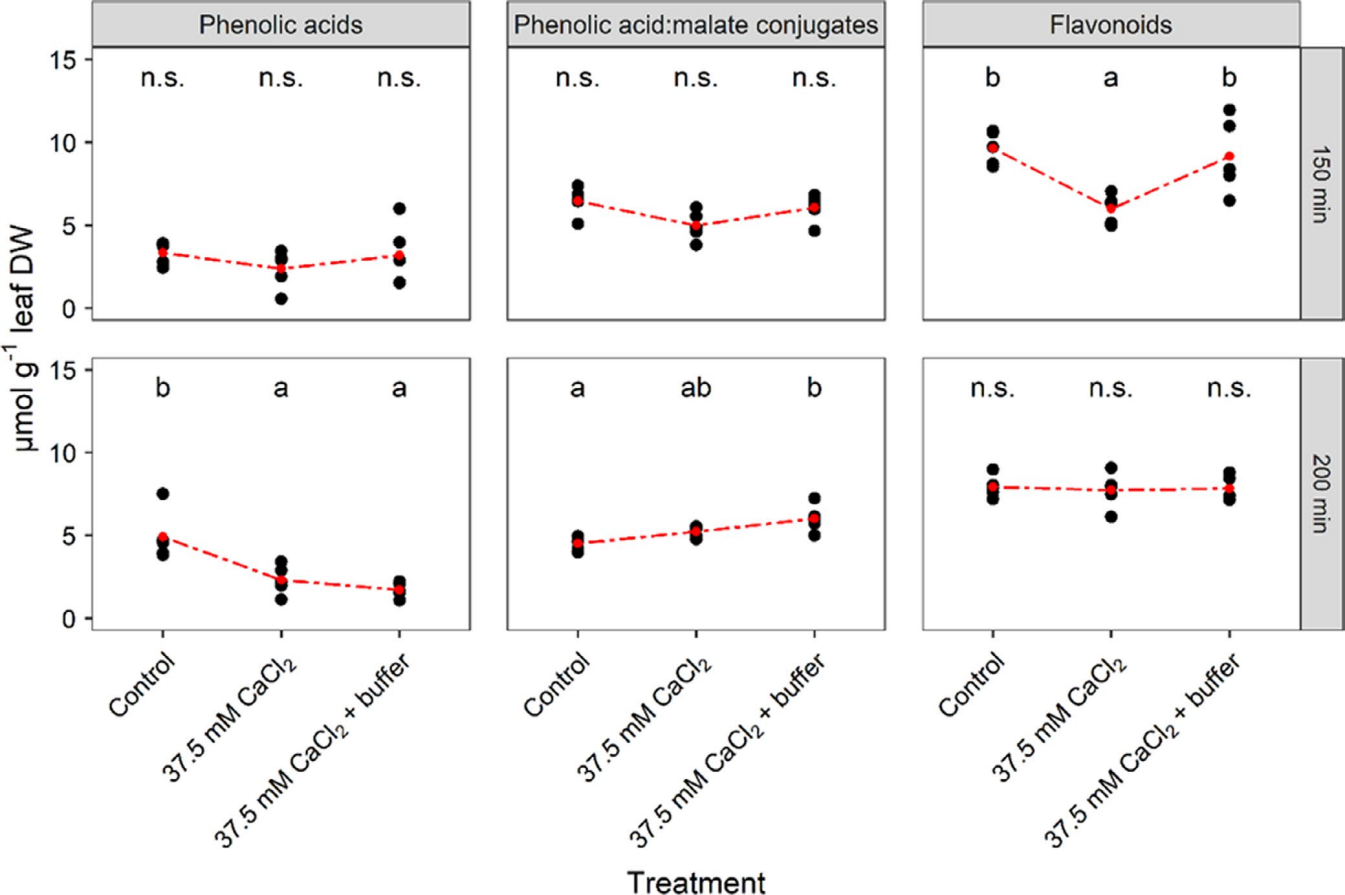
Salinity effects on total contents of phenolic acids, phenolic acid:malate conjugates and flavonoids. A univariate comparison between salt‐stressed Pak choi plants that were treated with 37.5 mM CaCl_2_ at the roots, equally salt stressed plants that had their pH_apo_ clamped in the acidic range by infiltration of 5 mM citric acid/sodium citrate (pH 3.6) and an unstressed control group. Samples were taken at 150 and 200 min after the onset of salt stress. Black circles: scatter plot; red circles/dashed line: interaction plot. Small letters indicate significant differences between treatment groups (Tukey‐HSD; α = 0.05; *n* = 5), "n.s." indicates the absence of a significant interaction.

HPLC analysis identified three flavonoids (FLs) and eight phenolic acid (PA) derivatives in leaves of Pak choi with contents ranging from 0.004 to 5.781 µmol/g DW (see Table 1). Similar content ranges of the single metabolites were recently validated for Pak choi (Heinze et al., 2018). Summing up the abundances of all three individual flavonoids (total FLs) revealed a significant decrease from 9.65 (±0.45 *SEM*) µmol/g leaf DW under control conditions to 5.99 (±0.40 *SEM*) µmol/g leaf DW under CaCl_2_ stress conditions 150 min after exposing roots to stress (Figure 2). Of note, this decrease in total leaf FLs was inhibited when the leaf apoplast of the CaCl_2_‐stressed plants had been clamped in the acid range prior CaCl_2_ addition. Both effects were not detectable after a further 50 min (e.g. at 200 min after CaCl_2_ addition).

**TABLE 1:** Contents of phenolic acid, phenolic acid:malate conjugate and flavonoid derivatives in Pak choi leaves under CaCl_2_ stress with or without clamping of leaf pH_apo_

Metabolite			150 min after CaCl_2_ addition µmol / g leaf DW						200 min after CaCl_2_ addition µmol / g leaf DW						One‐Way ANOVA (time point)	
			Control		37.5 mM CaCl_2_		37.5 mM CaCl_2_ + buffer		Control		37.5 mM CaCl_2_		37.5 mM CaCl_2_ + buffer		150 min	200 min
	Abbr	Class	mean	±SEM	mean	±SEM	mean	±SEM	mean	SEM	mean	±SEM	mean	±SEM		
Coumarylquinic acid	COA	PA	0.98	0.11	0.75	0.16	0.93	0.29	1.63^**b**^	0.22	0.69^**a**^	0.13	0.47^**a**^	0.07	n.s.	*
Caffeoylquinic acid	CQA	PA	1.88	0.15	1.28	0.31	1.80	0.49	2.77^**b**^	0.38	1.32^**a**^	0.22	0.98^**a**^	0.13	n.s.	*
Caffeic acid derivative	CAAD	PA	0.23	0.02	0.08	0.03	0.19	0.06	0.10	0.03	0.12	0.02	0.12	0.01	n.s.	n.s.
Feruloyl glucoside	FGL	PA	0.26	0.03	0.26	0.04	0.26	0.07	0.41	0.10	0.19	0.03	0.14	0.02	n.s.	n.s.
Hydroxyferuloyl malate	HFM	PAmC	0.60	0.05	0.43	0.08	0.52	0.07	0.37^**a**^	0.03	0.49^**b**^	0.02	0.56^**b**^	0.03	n.s.	*
Feruloyl malate	FMA	PAmC	2.23	0.14	1.60	0.08	1.98	0.16	1.34	0.18	1.88	0.07	2.15	0.25	n.s.	n.s.
Caffeoyl malate	CMA	PAmC	1.17	0.07	1.11	0.16	1.32	0.14	1.00	0.13	0.91	0.02	1.22	0.08	n.s.	n.s.
Sinapoyl malate	SMA	PAmC	2.45	0.16	1.85	0.14	2.26	0.08	1.82	0.14	1.96	0.09	2.08	0.10	n.s.	n.s.
Kaempferol‐3‐O‐hydroxyferuloyl diglucoside‐7‐O‐glucoside	KHDG	FL	1.78	0.15	1.23	0.09	1.74	0.17	1.25	0.08	1.54	0.11	1.59	0.10	n.s.	n.s.
Kaempferol‐3‐O‐caffeoyl diglucoside‐7‐O‐glucoside	KCDG	FL	3.85	0.40	3.08	0.14	4.04	0.25	3.58	0.38	3.22	0.27	3.60	0.13	n.s.	n.s.
Isorhamnetin‐3‐O‐glucoside	IRG	FL	4.02	0.66	1.68	0.19	3.38	0.71	3.09	0.42	2.96	0.30	2.64	0.24	n.s.	n.s.
Total Phenolic acids	PA		3.35	0.30	2.38	0.52	3.19	0.84	4.91**^b^**	0.67	2.33**^a^**	0.39	1.72**^a^**	0.21	n.s.	*
Total Phenolic acid:malate conjugates	PAmC		6.45	0.38	4.98	0.39	6.07	0.38	4.53**^a^**	0.19	5.23**^ab^**	0.15	6.01**^b^**	0.36	n.s.	*
Total Flavonoids	FA		9.65 **^b^**	0.45	5.99 **^a^**	0.40	9.16 **^b^**	1.01	7.93	0.30	7.73	0.48	7.83	0.33	*	n.s.

Note

Asterisks indicates mean differences (one‐way ANOVA; α = 0.05, *n* = 5) for the respective time point; "n.s." indicates the absence of a significant interaction. Small letters show significant differences between treatment groups (Tukey‐HSD; α = 0.05, *n* = 5). Abbr, Abbreviation of the structure assignment; PA, phenolic acid; FL, flavonoid, PAmC, phenolic acid:malate conjugate.

Of the eight PAs, four derivatives were identified as malate conjugates. Summation of the single metabolites of the free total phenolic acids (total PA) as well as the total phenolic acid:malate conjugates (total PAmC) revealed similar trends with respect to the CaCl_2_‐effect on FL contents, however, not being significant (Table 1). Yet, significant reductions in the total PA abundances were detected after 200 min. Thus, CaCl_2_ treatment decreased total PA contents from 4.91 (±0.67 *SEM*) µmol/g leaf DW in non‐stressed control plants to 2.33 (±0.39 *SEM*) µmol/g leaf DW. Clamping of the leaf pH_apo_ to pH 3.6 prior CaCl_2_‐treatment led to a further drop down to 1.72 (± 0.21 *SEM*) µmol/g leaf DW. Regarding the total PAmCs, contents of unstressed plants summed up to 4.53 (± 0.19 *SEM*) µmol/g leaf DW. CaCl_2_‐treatment increased the PAmC content to 5.23 (±0.15 *SEM*) µmol/g leaf DW, though this was not significant. A significant increase to 6.01 (±0.36 *SEM*) µmol/g leaf DW could only be determined after 200 min of CaCl_2_ salinity under simultaneous buffering of the pH_apo_ in the acidic range.

The comparison between the grouped metabolites revealed both a CaCl_2_ stress and a pH_apo_‐effect on the FL derivatives at 150 min after initiation of stress treatment (Figure 2). However in contrast, analyses of single PA derivative abundances, revealed stress effects only 200 min after the initiation of salt stress (Table 1). Compared to the non‐CaCl_2_‐treated control, the 200‐min treatment of plant roots with 37.5 mM CaCl_2_ caused a significant decrease of the PAs caffeoylquinic acid (CQA) and coumarylquinic acid (COA) from 2.77 (±0.38 *SEM*) to 1.32 µmol/g DW (±0.22 *SEM*) and from 1.63 (±0.22 *SEM*) to 0.69 µmol/g DW (±0.13 *SEM*), respectively. In relative terms, the CaCl_2_ stress resulted in less than 50% of the contents of CQA (‐52%) and COA (‐58%). In contrast, hydroxyferuloyl malate (HFM) contents increased significantly about 1.32‐fold from 0.37 (±0.03 *SEM*) to 0.49 µmol/g DW (±0.02 *SEM*) due to the CaCl_2_ salinity treatment. Statistical analysis did not indicate any significant effects for the three FLs kaempferol‐3‐O‐hydroxyferuloyl diglucoside‐7‐O‐glucoside (KHDG), kaempferol‐3‐O‐caffeoyl diglucoside‐7‐O‐glucoside (KCDG), or isorhamnetin‐3‐O‐glucoside (IRG). Buffering the apoplast at a pH of 3.6 prior to CaCl_2_ treatment had no effect on the content of the individual secondary metabolites.

## 4 DISCUSSION

According to Lager et al. (2010), changes in the external pH rapidly alter expression of many genes as shown for roots of *Arabidopsis thaliana*. In agreement with these results, we have found that the chloride‐inducible transient increase of the leaf apoplastic pH provoked an increase of leaf protein abundances (Geilfus et al., 2017). Among these proteins, key enzymes of the branched phenylpropanoid pathway were found. Thus, the question arose, whether congruent changes arise on the level of the metabolome, especially of phenolic plant metabolites in response to a chloride‐induced transient alkalinization of pH_apo_.

In the presented study, real‐time *in‐planta* pH monitoring was used to demonstrate that the leaf pH_apo_ of Pak choi can also be modulated by means of chlorine salinity (Figure 1; black kinetic). Previous studies with chloride‐accompanying counterions revealed that the transient pH‐increase is attributable to the anion (i.e. Cl^−^) and not to the accompanying cation or the osmotic compound of the stress treatment (Geilfus & Mühling, 2013). The transient increase in pH_apo_ that followed the CaCl_2_‐treatment could be inhibited by clamping of the apoplast in the acidic range. Both groups (37.5 mM CaCl_2_ vs. 37.5 mM CaCl_2_ + pH buffer) accumulated Cl^−^ contents which were far above the requirements for chlorine as a micronutrient (Geilfus, 2018), indicating the beginning of excessive exposure of the tissue to Cl^−^.

Under conditions of salinity, evidence for a diminishing effect on phenolic levels was found for sugar cane (Wahid & Ghazanfar, 2006), red pepper (Navarro et al., 2006), lettuce (Blasco et al., 2013) and broccoli leaves (López‐Berenguer et al., 2009). López‐Berenguer et al. (2009) placed a spotlight on tissue differences because salinity increased phenolics in the florets of broccolis while overall tissue concentration decreased. Other salt stress studies reported increasing shoot content of phenolics in cotton (Wang et al., 2015), buckwheat (Lim et al., 2012), broad bean (Dawood et al., 2014), safflower (Gengmao et al., 2015), radish sprouts (Yuan et al., 2010), and the halophytic sea rocket (Ksouri et al., 2007). Additional beneficial effects of salinity on phenylpropanoid quantities were recently described for several species of the *Brassicaceae* family (Linić et al., 2019). Accordingly, Yun et al. (2016) reported elevated FL and phenol contents as well as an increased radical scavenging activity in salt‐stressed Pak choi.

Results of the present study on the fast responses of chlorine salinity on PA and FL derivatives in leaves of Pak choi contribute novel information on this topic. Accumulation of phenylpropanoids as reported by Yun et al. (2016) were not yet observed. In contrast, it was demonstrated that 150 min after the onset of salt stress, total FLs decreased significantly (Figure 2). A reduction in the total PAs and simultaneous increase in total PAmC abundance could only be detected, 200 min after CaCl_2_‐treatment. The inhibition of the CaCl_2_‐induced transient increase in the leaf pH_apo_, achieved by fixing the apoplastic pH in the acidic range, resulted in total FL contents equal to the unstressed control plants (Figure 2). This is the first proof that early chlorine salinity‐specific effects on the leaf FL pattern are controlled by the leaf pH_apo_, although the physiological significance of such a pH‐dependency remains elusive. A further new aspect of this study is that the phenolic subgroups total PAs and total PAmCs segregated in terms of abundance under conditions of salinity. In contrast to the effects on total FLs, this salinity effect was not exerted by the salt stress‐induced transient alkalinization of the leaf pH_apo_. This implies that different salinity‐induced mechanisms are relevant for shaping the two metabolic profiles. Nonetheless, these results showed that the pH of the leaf apoplast is a factor that links salinity with leaf metabolic responses. Previous studies have shown that this pH‐effect is attributable to the anion, that is, Cl^−^ and not related to the cation, that is, Ca^2+^ (Geilfus & Mühling, 2013).

However, comparison of the single classes of metabolites revealed that by far not all of the analysed derivatives of PAs and FLs are controlled by the leaf pH_apo_ (Table 1). When compared to non‐stressed Pak choi leaves, salt stress caused statistically significant changes, not related to or reversed by the pH_apo_. The PAs COA and its metabolic successor CQA were reduced by CaCl_2_‐salinity. Both metabolites, particularly CQA, are key substrates that act as precursors in the phenylpropanoid pathway, therefore being educts for the synthesis of CQA derivatives as well as for lignin synthesis (Menin et al., 2010).

Thus, the changes observed may indicate a modulation of the phenylpropanoid metabolism, that is, towards the synthesis of cell‐wall compounds. Similar implication have been reported for short‐term salt stressed *A. thaliana* and *Oryza sativa* plants (Kim et al., 2007; Wang et al., 2009). Of note, cell‐walls are stiffened by salinity (Geilfus et al., 2017; Neumann et al., 1994). Among the metabolites, only HFM showed a significant increase in abundancy, being detected at 200 min after the onset of salt stress. HFM is a PA that is argued to be an antifungal agent (Quentin et al., 2014) and was found to be increasingly synthesized in three different *Brassica rapa* cultivars, upon fungal infection (Abdel‐Farid et al., 2009). Here, we found an HFM accumulation provoked by abiotic stress. Further insights on the physiological and biochemical role of HFM as well as the PAmCs in general, is scanty. Therefore, implications for functional traits linked to an accumulation of PAmC are hardly possible.

The contents of IRG, KCDG, and KHDG were not influenced by salinity. Similar to the marked dynamic of total FLs, IRG, a major constituent of the phenolics in *Brassica* (Romani et al., 2006), decreased by 58%, 150 min after the addition of 37.5 mM CaCl_2_ to the roots. Yet, due to great variances in the control and fixed‐pH salt stressed treatment group, no significant effect could be deduced.

## 5 SUMMARY

The aim of this study was to evaluate the role of pH of the leaf apoplast in modulating the abundances of derivatives of PAs and FLs in leaves of Pak choi under chlorine salinity. Starting point was that our previous studies revealed chlorine salinity‐induced changes in the pH of the leaf apoplast modulating abundances of two key enzymes of the phenylpropanoid biosynthesis, viz. PAL and cinnamyl alcohol dehydrogenase. Here, we provided evidence for early (minutes) salinity‐specific reductions of the abundance of plant PAs and FLs, which were mediated by pH_apo_. This is the first evidence that salinity‐induced changes of the leaf apoplastic pH are functional with respect to the modulation of metabolite abundances. Additionally, PAs that are conjugated to malate were affected in a reciprocal way by salt when compared to non‐malate conjugates. This implies that these PAmCs comprise a salt‐responsive subgroup of phenolics, an observation that, to our best knowledge, had never been reported before.

## CONFLICT OF INTEREST

Authors have no conflict of interest to declare.

## AUTHOR CONTRIBUTIONS

C.M.G. conceived and planned the experiments. C.M.G. and N.F. performed the analysis. P.M. performed the statistical analysis and designed the figures. P.M. took the lead in writing the manuscript with C.M.G. Manuscript was written with input from all authors. All authors contributed to the interpretation of the results.
